# The (r)evolution of chemical space and molecular modeling: a time-resolved perspective

**DOI:** 10.1007/s10822-026-00850-1

**Published:** 2026-06-01

**Authors:** Aylin Del Moral-Morales, Francisco L. Feitosa, Carolina Horta Andrade, José L. Medina-Franco

**Affiliations:** 1 DIFACQUIM Research Group, Department of Pharmacy, School of Chemistry, Universidad Nacional Autónoma de México, Avenida Universidad 3000, Mexico City, 04510 Mexico; 2 Departamento de Ciencias Naturales, Universidad Autónoma Metropolitana-Cuajimalpa (UAM-C), Vasco de Quiroga 4871, Mexico City, 05348 Mexico; 3 Laboratory for Molecular Modeling and Drug Design (LabMol), Faculty of Pharmacy, Universidade Federal de Goiás, Rua 240, 406, Goiânia, Goiás 74605-220 Brazil

**Keywords:** Artificial intelligence, Chemical space, Chemoinformatics, Chemical libraries, Computational chemistry, Databases, Molecular modeling, Structure–property associations—SPA

## Abstract

The number and types of chemical compounds are expanding at an unprecedented rate. To model existing chemicals and aid in the design of novel chemical structures, appropriate computational approaches, tailored to the goals of specific projects, have evolved over time. This review analyzes the expansion of “chemical space” by tracing the historical milestones that have shaped molecular modeling from its inception to the present day. Utilizing data from public compound databases and a systematic bibliometric analysis of peer-reviewed literature, including specialized sources like the *Journal of Computer-Aided Molecular Design*, we mapped the co-evolution of chemical data and the algorithms designed to process it. While drug discovery has historically been the primary driver of this growth, our discussion extends to other domains, including macromolecular structural space. Due to the nature of public data, this analysis focuses mostly on open-access repositories with a few mentions of proprietary industrial libraries. By examining the trends in publications, this article provides a perspective on the current state of the field and the future trajectories of molecular modeling in an era of big data and artificial intelligence.

**Graphical abstract:**

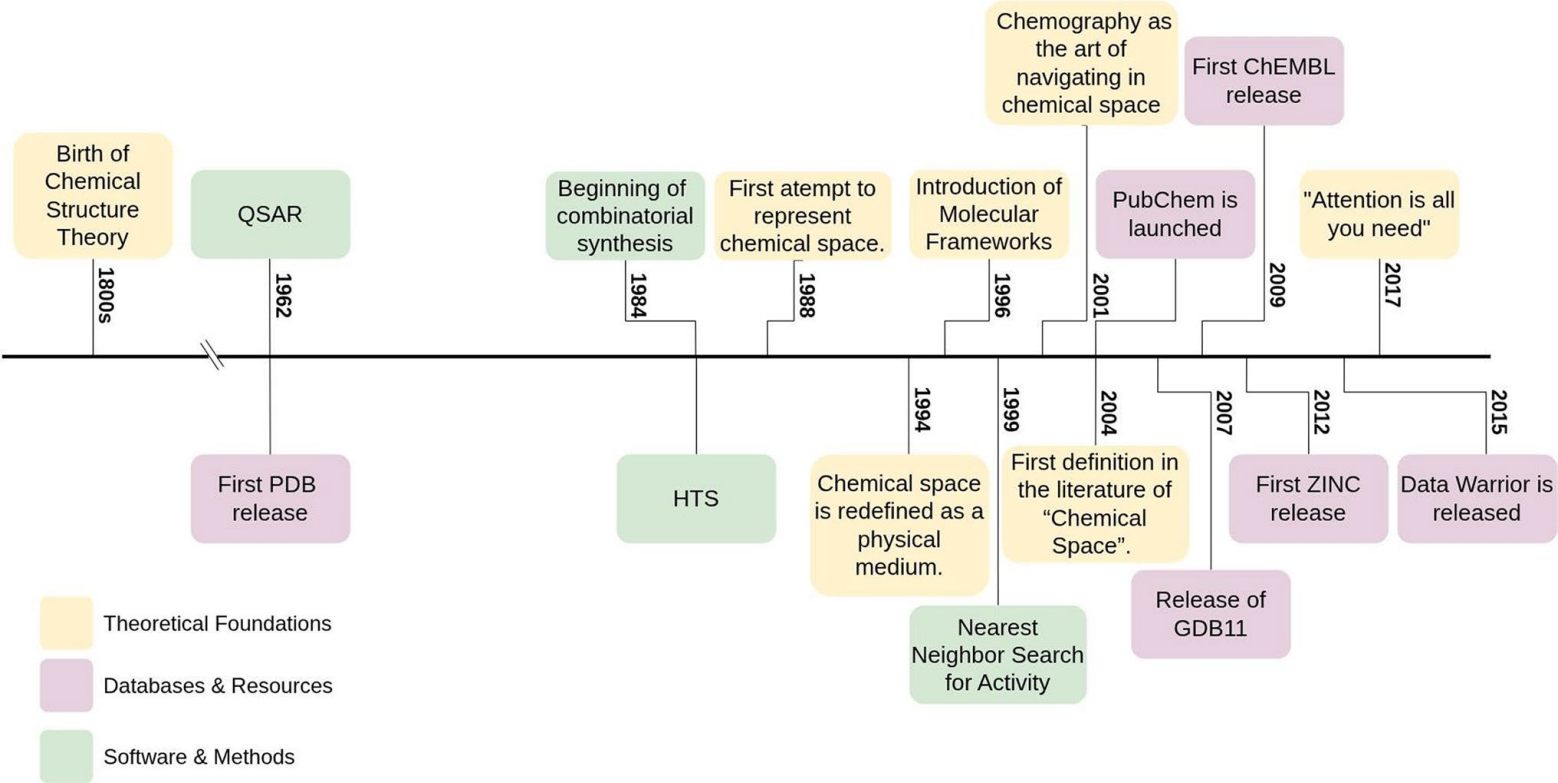

**Supplementary Information:**

The online version contains supplementary material available at 10.1007/s10822-026-00850-1.

## Introduction

The current notion of “chemical” space albeit with different formal definitions, has helped the scientific community gain a sense of the number of chemicals that exist or potentially can exist, along with their relationships [1]. The specific study of each type of chemical varies with the chemistry subfield, so that, in most practical applications, the concept of chemical subspaces is used. Herein, we emphasize that although drug discovery research has and continues to drive the growth of the number of compounds, in particular, small organic molecules, the chemical space, in its broad sense, involves every single existing or potential chemical compound, regardless of the specific application domain.

Since the existing chemical space is so vast and continues to expand, several questions emerge that could resemble questions that one can ask in parallel with the cosmic universe. For example, how was the chemical space at the beginning; how fast is it growing, quoting Cherkasov [2]? How has it been evolving over time and in what directions? How is the explored chemical space expected to be in the near and distant future?

Alongside the number and type of chemicals broadly used and heavily studied (as reported in peer-reviewed journals and public databases), questions also emerge, such as what computational approaches have been used to design and model chemical compounds? What are the milestones in the history of molecular modeling? Of course, it is anticipated that the methods themselves will have to adapt to the specific goals of the molecular modeling projects (including the specific areas of application for instance drug discovery, chemical synthesis and catalysis, inorganic chemistry and so on), the level of the expected precision of the modeling, the types and sizes of molecules under study, and the number of chemical compounds under study. In parallel the comparatively lower cost of computational methods relative to traditional wet lab experiments, which positions them a cost-effective complement to experimental workflows by enabling large-scale virtual screening and reducing the number of compounds that require costly synthesis and biological testing, is also a key factor in the growing adoption and development of these approaches.

Thought-provoking questions regarding the history of compound libraries, computational methods, and their implementation (driven hand in hand by the evolution of software) have led to several types of analyses. Indeed, there are recent publications, including reviews, perspectives, and opinion papers that have discussed the evolution and growth of chemicals and chemical libraries. For example, Lopez-Perez et al. [3] conducted an analysis using similarity-based metrics (using instant Similarity—iSIM) [4] and clustering approaches (BitBIRCH) [5], enabled the systematic evaluation of how the growth of large chemical libraries relates to structural diversity, and demonstrating that an increase in the number of compounds does not necessarily translate into a proportional expansion of diversity. Moreover, Llanos et al. [6] employed large-scale reaction data mining to reconstruct the temporal evolution of chemical space, providing a historical perspective on how chemical transformations drive changes in its composition over time. Leal et al. [7] investigated the emergence of structural patterns within early chemical space, using relational and historical analyses to show how fundamental periodic relationships were implicitly encoded prior to the formal development of the periodic system. In 2022, Restrepo [8] discussed the theoretical limits and evolution of chemical space, highlighting its enormous size and the challenges associated with its representation and storage.

In terms of analysis discussing trends of modeling techniques, Sheikhpour and Gharaghani [9] recently traveled through the early foundations of Quantitative Structure–Activity Relationship (QSAR) and explored the more advanced methodologies that accompanied the development of the hardware, such as deep learning and graph neural networks. Another notable review by Mao et al. [10] provides a comprehensive overview of machine-learning-based QSAR strategies, highlighting the transition from descriptor-driven and traditional statistical models to data-intensive frameworks that integrate deep learning, multi-omics data, and simulation techniques such as molecular dynamics.

The shift from predictive to generative modeling in cheminformatics gained traction with Olivecrona et al. [11, 12], who combined recurrent neural networks with reinforcement learning to generate molecules with target properties. The work showed that generative artificial intelligence (AI) could reach regions of chemical space that traditional virtual screening approaches do not typically access.

More recently, Gao et al. [13], addressed a persistent limitation of generative models: the tendency to produce molecules that are novel but impractical to synthesize. Their framework embeds synthetic feasibility directly into the generation process, rather than filtering for it afterward.

Despite recent reviews covering parts of the history of chemical space and computational methods having been published, a combined analysis of the growth and evolution of chemical data sets and libraries, along with molecular modeling methodologies and peer-reviewed publications, is missing. Herein, we discuss a time analysis of the growth in the number and types of chemical compounds, as well as the evolution of commonly used computational strategies that have shaped the design and modeling of chemical space over time. The discussion is grounded on a systematic analysis of the peer-reviewed literature from 1976 to date. The manuscript is organized into six sections. After this Introduction, Sect. “Evolution of the definition of chemical space” discusses a time analysis of the chemical space as a concept e.g., the history of the semantics and the use of the idea or concept of “chemical space” and related terms. Sect. “Evolution of computational methods and strategies” discusses an analysis of the evolution of computational methods and strategies. The next section presents an updated and general survey of the change of the number of chemicals over time and rate of growth discussing small- and biomacro- molecules. The discussion is based on public data taking as a proxy two major public compound databases. Since the discussion is based on public data, several chemical libraries available only to the private sector are not discussed in detail. Sect. “Journals chronicling the development of chemical space and molecular modeling” discusses exemplary peer-reviewed journals that have been documenting the evolution of the chemical space and computational methods. Sect. “Conclusions and future directions” presents summary conclusions and future directions.

## Evolution of the definition of chemical space

The concept of space has fascinated scholars for centuries and has evolved alongside advances in mathematics and science. Early ideas about space can be traced back to Aristotle, who around 300 BC proposed that space could have no more than three dimensions and conceived it as existing only in relation to objects embedded within it [14]. A major shift occurred in the seventeenth century with the development of analytic geometry by Descartes, which enabled points to be described in Euclidean space [15]. This mathematical framework later allowed the formal development of higher-dimensional spaces by Schläfli and Riemann in the nineteenth century, providing the foundations for modern multidimensional representations.

In mathematics, spaces are often defined as sets of objects together with relations among them, while subspaces correspond to subsets that retain the same relationships. In coordinate-based representations, objects are described by vectors of properties that define their positions in multidimensional spaces [16]. Distances between objects can then be interpreted as measures of similarity or dissimilarity [17]. When applied to chemistry, this framework enables molecules to be organized within property- or structure-based spaces. In these representations, molecules with similar descriptors tend to occupy nearby regions [18].

The concept of chemical space is of chemical space is currently broadly used in several areas of chemistry, with particular emphasis in pharmaceutical and organic chemistry. Also, it is a core concept in chemoinformatics and central to computer-aided molecular design [19]. However, a vital distinction must be made between theoretical and empirical definitions. In theoretical chemistry, compound chemical space (CCS) is defined as the exhaustive, static collection of every feasible, metastable atomic configuration permitted by the laws of physics [20]. From this fundamental perspective, the ‘universe’ of possible matter is pre-defined. In contrast, this review focuses on the Informatics Chemical Space (ICS), the dynamic, ever-expanding subset of the theoretical universe that has been cataloged, synthesized, or rigorously characterized through computational prediction. While the theoretical boundaries are fixed, the ICS expands as synthetic and algorithmic advancements allow researchers to map previously ‘dark’ regions of the molecular landscape.

Within this conceptual framework, chemical space can be defined as the set of all known molecules together with all hypothetical molecules that satisfy chemical and valence rules. Despite its enormous size, chemical space can be partitioned into numerous overlapping chemical subspaces, often associated with specific compound classes or research domains such as organic, pharmaceutical, material science, or biochemistry. Of note, the application or extension of chemical space to analyze biomolecules can lead to the notion of biological space (which could be a more appropriate term used in the discipline of bioinformatics). Of note, chemical space is an abstract concept; in practice, researchers analyze computational representations of chemical space derived from specific descriptors, similarity metrics, and visualization techniques.

However, it is not straightforward to trace back and identify with absolute certainty the first time it was used in the open literature as it is used today. There are a few attempts to revise the definitions of chemical space proposed in the literature. For example, eight definitions published in the literature between 2004 and 2021 have been collected in [21]. Hereunder, we discuss more in depth the origins and evolution of the definition of chemical space.

Table 1 summarizes a timeline with representative milestones that have shaped the concept of chemical space and associated terms. The table summarizes the fact (key event or milestone), who (researcher or institution), and when (year) the milestone took place. The timeline is not exhaustive but is intended to exemplify key steps in the background, setting, and development of chemical space.

**Table 1:** Relevant events associated with chemical space definition, and exploration

Year	Event	Researcher or Institution	References
Middle 1800s	Chemical structure theory: chemical properties of a molecule depend on the number of atoms and the structure	Alexander M. Butlerov	[22]
1962	QSAR is established	Hansch et al.	[36]
1962	First Protein Data Bank (PDB) release with 13 protein structures	Brookhaven National Laboratory	[37]
1984	Beginning of combinatorial chemistry (peptide synthesis)	Geysen, Meloen and Barteling	[38]
1984	Introduction of high-throughput screening (HTS) methodology	Pfizer	[39]
1988	“This paper is one of the first attempts to define chemical structure space for a large universe of chemicals.” The authors present a three-dimensional reduction of chemical structures	Veith et al.	[28]
1988	Simplified Molecular Input Line Entry System (SMILES) are proposed	Weninger	[30]
1992	Description of several files used to represent molecule structure such as molecule (MOL) and structure data files (SDF)	Molecular Design Limited	[31]
1994	The authors establish a correlation between molar volume and thermodynamic parameters, specifically the enthalpies of formation and vaporization. Chemical space is redefined as a physical medium where volume is intrinsically linked to the mass, energy, and electromagnetic properties of the constituent atoms	Gutman,Fishtik and Nagypál	[40]
1996	First proposition for the use of Molecular frameworks	Bemis and Murcko	[41]
1998	HTS is used to describe the fingerprints associated with a better binding affinity	Kauvar et al.	[42]
1999	Suggestion that finding the nearest similar neighbour could expand the possibilities of finding active compounds	Stanton et al.	[43]
2000	Presentation of an approach to find useful structural descriptors for molecule clustering	Taraviras, Ivanciuc and Cabrol-Bass	[44]
2001	A novel method (called LaSSI) for computing chemical similarity from chemical substructure descriptors is described	Hull et al.	[45]
2001	Definition of Chemography as the art of navigating in chemical space	Oprea and Gottfries	[46]
2001	Ftrees-FS, an algorithm for similarity searching in large chemical spaces	Rarey and Stahlb	[47]
2004	Horizon Symposium on ‘Charting Chemical Space: Finding New Tools to Explore Biology.’	Nature Publishing Group and Aventis (now known as Sanofi)	[48]
2004	First formal definition in the literature of “Chemical Space.”	Kirkpatrick and Ellis	[32]
2004	PubChem and Chemical Entities of Biological Interest (ChEBI) are Launched	US National Institutes of Health and European Bioinformatics Institute	[49]
2006	FlexNovo, a structure-based molecular-design software that searches large fragment spaces, is released	Degen and Rarey	[48]
2007	REAL Database is launched with 29,215,900 synthetically feasible compounds	Enamine	[50]
2007	Release of GDB11, a theoretical database with all molecules of up to 11 atoms of C, N, O, and F possible. It contains 26.4 million molecules	Fink and Reymond	[51]
2009	First ChEMBL release, a database of bioactive compounds	European Bioinformatics Institute	[52]
2012	First ZINC release, a database for commercially available compounds	University of California San Francisco	[53]
2012	Release of GDB-17, a database enumerating all possible molecules with 17 atoms of C, N, O, S, and halogens, containing 166.4 billion organic molecules	Ruddigkeit et al.	[54]
2015	Data Warrior, a software for the exploration of chemical space, is released	Sander et al.	[55]
2017	Publication of the article “Attention is all you need” about machine learning	Vaswani et al.	[56]
2020	Release of Synthetically Accessible Virtual Inventory (SAVI) a database of over 1 billion compounds predicted to be easily synthesizable	Patel et al.	[57]
2021	Introduction of SpaceMACS, a tool for Maximum subspace searching	Schmidt et al.	[58]

Since Alexander Butlerov suggested in the nineteenth century that the chemical properties of a molecule depend on the number of atoms and the structure [22], there have been several attempts to describe such relationships. Although the phrase “chemical space” appeared sporadically in 1960s literature, these early mentions typically referred to the behavior of molecules in physical outer space [23–25]. It was not until the 1980s that the term was formally adopted as a conceptual framework to describe molecular behavior in chemical reactions [26, 27]. This era marked a shift in the field, witnessing the emergence of foundational databases and methodologies for systematic exploration. Key developments included Quantitative Structure–Activity Relationships (QSAR), the implementation of High-Throughput Screening (HTS), and the establishment of the Protein Data Bank (PDB), which remains one of the world’s premier repositories for macromolecular structural data.

It was in 1988 that Veith et al. published one of the first documented attempts to define “chemical structure space for a large universe of chemicals” [28]. In this study, the authors present a visualization of 19,972 molecules using descriptors of chemical structure and dimensional reduction through principal components analysis. Veith, the publication’s first author, later founded the International QSAR Foundation and created the QSAR Toolbox, a computational tool to assess chemical toxicity [29].

Parallel to these conceptual advancements, the late 1980s saw the standardization of molecular file formats essential for modern computational chemistry. The introduction and formalization of the Simplified Molecular Input Line Entry System (SMILES), along with MOL and Structure Data Files (SDF), revolutionized the field [30, 31]. These lightweight, machine-readable representations bypassed the storage constraints of earlier formats, thereby enabling the creation of expansive molecular repositories and the implementation of high-throughput computational workflows.

However, the first formal definition of the “chemical space” itself, was published in the literature by Kirkpatrick and Ellis in 2004 as an introduction to the Horizon Symposium on ‘Charting Chemical Space: Finding New Tools to Explore Biology’ organized by the Nature Publishing Group and the pharmaceutical company Adventis [32, 33]. This symposium seems to be a turning point, establishing “chemical space” as the primary descriptor for the vast universe of small-molecules. By late 2004, a series of seminal articles were published, effectively standardizing the term and providing the basis for the systematic exploration of chemical diversity [32, 34, 35].

As discussed in the preceding paragraph, since then, there are several other formal definitions of chemical space provided in the literature and reviewed recently [21]. As analyzed in the next section, the “chemical space” term has been used as a keyword in peer-reviewed publications.

Following the formal adoption of “chemical space” as a foundational concept, several databases were established to systematically catalog and annotate both known and theoretical molecular territories. In 2004, PubChem and Chemical Entities of Biological Interest (ChEBI) were launched, providing high-capacity, open-access repositories for small molecules and bioactive entities [49]. Simultaneously, the GDB-11 database was introduced, marking one of the first major efforts to map the “exhaustive” theoretical space by enumerating all 26.4 million possible organic molecules containing up to 11 atoms of C, N, O, and F [51]. Also, in 2007, the Enamine REAL database was introduced; the first version contained 26 million compounds with drug likeness properties and available for synthesis upon request, which represented a milestone for synthetic chemistry [50]. Currently, the database holds 13.6 billion compounds [59] and is a valuable resource for high-throughput screening. A more recent example is the Release of the Synthetically Accessible Virtual Inventory (SAVI), a database with 1 billion compounds predicted to be easily synthesized by two-reactant syntheses [57].

The landscape of bioactivity data was further transformed in 2009 with the inaugural release of ChEMBL, which offered a curated, large-scale repository of molecules with associated pharmacological properties [52, 60]. By 2012, the ZINC database was launched, specifically catering to the needs of virtual screening by indexing millions of commercially available compounds [53].

Notably, one of the most highly cited publications within the “chemical space” keyword is the 2014 introduction of DataWarrior, an open-source platform for the visualization and analysis of chemical datasets [55]. Finally, any discussion of the modern expansion of chemical space must acknowledge the seminal 2017 paper, “Attention Is All You Need” [56]. As will be detailed in the following section, this contribution has become the foundation for contemporary machine learning models, enabling the generative exploration of theoretical chemical space.

## Evolution of computational methods and strategies

Over the years, the type and number of computational methods have evolved, aiming to capture, depending on the area of application, the essential requirements of chemical compounds to address certain goals or unmet needs. In this section, we first discuss the evolution of computational methods from 1995 to 2025 and then the new trends in computational methodologies, with special focus on the generation of virtual libraries and the computational methods supporting the development of automated libraries.

### Computational methodologies to model existing chemical compounds

To explore the evolution of computational methodologies over time related to chemical space, we conducted a search in Web of Science using the term “chemical space,” which, excluding review articles, returned 5861 publications spanning from 1960 to 2025. The temporal distribution of these publications is presented as a histogram in Figure S1A.

**Fig. 1:**
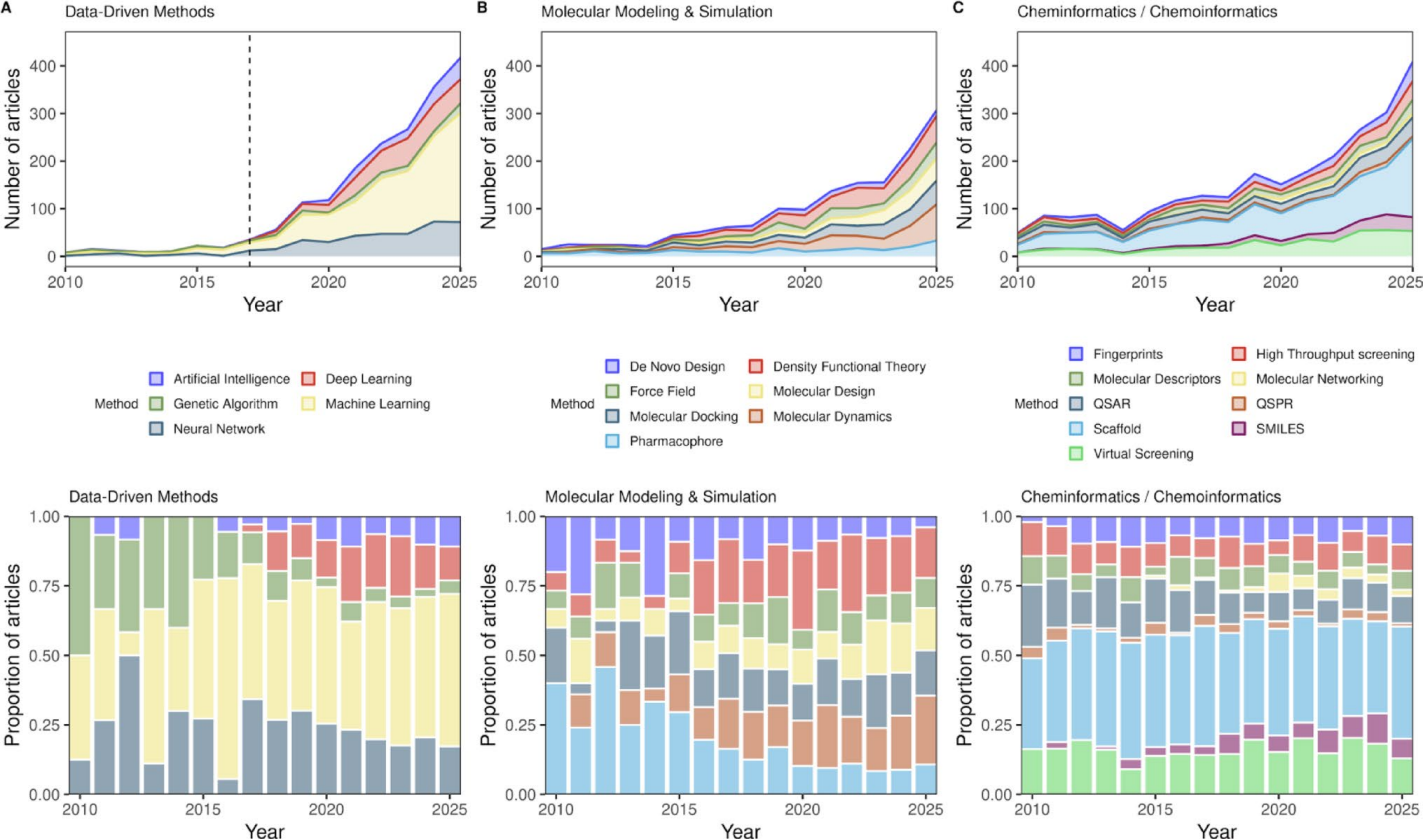
Annual publication trends for articles indexed with keywords related to **A** Data-Driven Methods (the dashed line indicates the publication of “Attention Is All You Need” in 2017), **B** Molecular Modeling and Simulations, and **C** Cheminformatics/Chemoinformatics. The upper panel shows the number of articles published with each keyword, from 1995 to 2025, and the lower panel shows the proportion of articles published each year

The collected metadata were then analyzed using VOSviewer [61] to perform bibliometric analyses and generate a keyword co-occurrence network, filtered for terms related to computational methodologies, as shown in Figure S1B. For clarity and improved visualization, the keywords were classified into four categories: Data-Driven Methods, Molecular Modeling & Simulation, Cheminformatics/Chemoinformatics, and Synthetic Chemistry. Of note, a recent bibliometric analysis of peer-reviewed papers indexed in PubChem showed that both terms “cheminformatics” and “chemoinformatics” are used almost indistinctively [62]. While the final category is addressed in a subsequent section, the first three were analyzed according to their temporal distribution (Fig. 1A–C). The analysis focuses on the period from 2010 to 2025, as this timeframe marks the accelerated growth phase for publications in these fields. For a comprehensive overview, the full publication timeline is provided in Figure S2.

Together, these observations suggest that the study of chemical space has evolved in parallel with advances in computational methodologies. While early approaches were predominantly centered on molecular modeling and simulation techniques, the increasing use of data-driven and cheminformatics-based methods reflects a shift toward the analysis and exploitation of large-scale chemical datasets.

This evolution is consistent with the broader adoption of the concept of “chemical space,” which began gaining prominence in the early 2000s, as indicated by Ngram analyses using Google Ngram Viewer [8]. The growing use of this term likely reflects both the field’s conceptual maturation and the increasing interest in systematically exploring chemical diversity.

Subsequently, this conceptual expansion was enabled by the steady increase in computational power, historically described by Moore’s Law [63], which allowed the handling of increasingly complex calculations and larger chemical libraries. In parallel, the expansion of publicly available chemical and biological datasets further facilitated the adoption of data-driven approaches (see Sect. “Change in the number and rate of chemicals”), as well as the development of more efficient molecular structure files and formats that reduced the memory requirements for computational workflows [30, 31].

More recently, particularly from around 2020 onwards, a sharper increase in data-driven methodologies can be observed. This trend likely reflects a combination of factors, including advances in machine learning architectures such as the introduction of the seminar paper “Attention is all you need” (which introduced the concept of Transformer-based architectures and revolutionized the industry) [56], improvements in hardware, and the rapid expansion of AI applications in drug discovery [64]. Additionally, the COVID-19 pandemic has been associated with a substantial increase in scientific output across disciplines, further contributing to the growth in publications during this period [65, 66].

Given that chemical space is closely associated (although not exclusively) with drug discovery, the role of computational chemistry methods in drug discovery has steadily increased. The excitement around AI methods, together with AI-based drug discovery companies, has accelerated the development of computer-aided molecular design methods. This is particularly evident in studies leveraging virtual screening, molecular modeling, and data-driven approaches to accelerate hit identification and optimization.

Importantly, the rise of data-driven approaches does not replace traditional molecular modeling strategies but rather complements them, enabling hybrid workflows that integrate structure-based methods with statistical and machine learning techniques. This convergence has become particularly relevant in the field, where the efficient exploration of chemical space remains a central challenge.

### Approaches to augment the size of chemical space

It can be argued that there are two types of chemical space: the known and the unknown. As we will discuss in the next section, although the known chemical space for small molecules is vast, it is not close to the total estimate of possible compounds. A rough estimate has been provided by Bohacek et al. who proposes that the number of molecules containing up to 30 atoms of C, N, O or S are likely more than 10^60^ [67, 68]. Other approximations mention that the possible number of compounds with a molecular weight lower than 1 kDa and containing only C, N, O, P, S or halogens could be up to 10^200^ [8, 69]. This means that the unexplored chemical space is a promising field; in fact, the discovery and production of new molecules is a key focus of many industries, such as the pharmaceutical, environmental, and agrochemical sectors.

As discussed above, the development of data-driven techniques for molecular modeling has pushed forward the generation of new molecules. Recent years have witnessed the appearance of ultralarge libraries for small compounds, mostly for drug discovery applications [70]. However, the theoretically generated molecules must be synthesized and analyzed in order to validate their predicted properties [13]. Here, we discuss the methodologies commonly used to synthesize and augment the chemical space, as reported in the peer-reviewed literature.

**Fig. 2:**
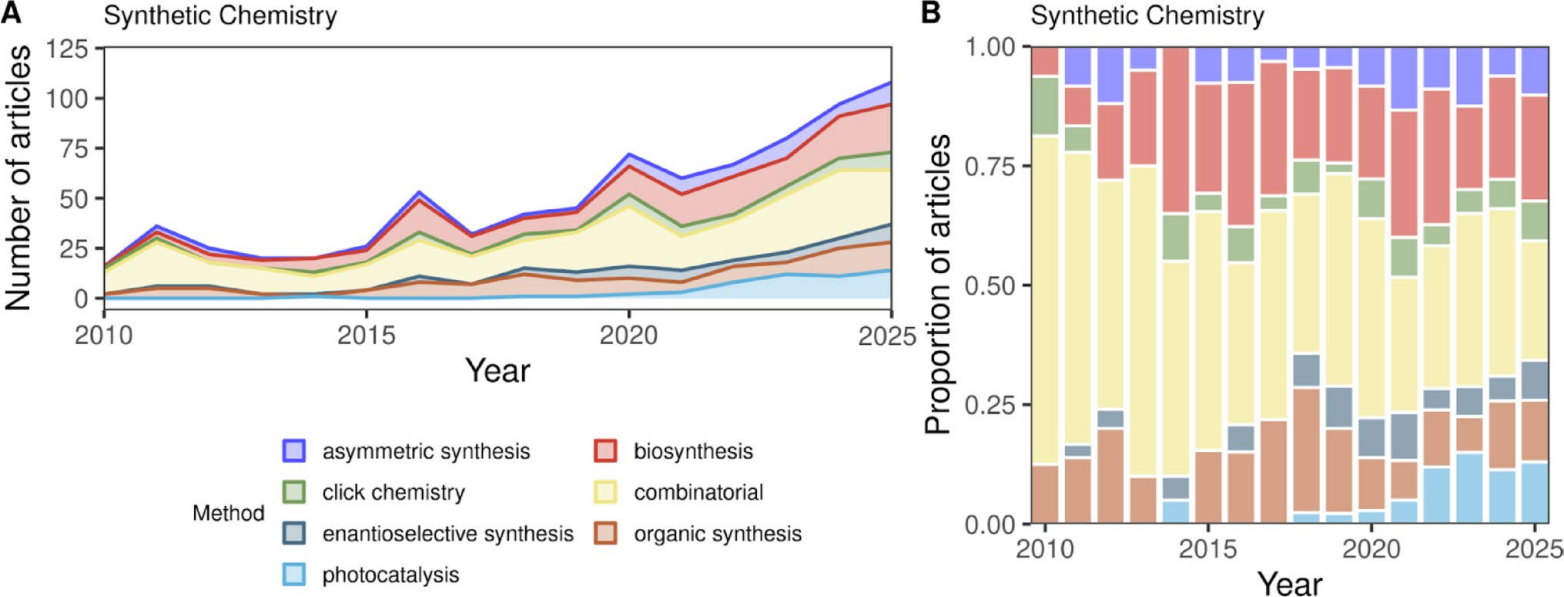
Annual publication trends from 2010 to 2025 for peer-reviewed articles related to chemical space and indexed with keywords related to Synthetic Chemistry. **A** The number of articles published with each keyword. **B** The proportion of articles published each year

Figure 2 illustrates the temporal evolution of synthetic methodologies cited in literature associated with chemical space. The timeline originates in the 1990s, dominated by techniques in organic synthesis and the emergence of photocatalysis. By utilizing light as a primary energy source for electron transfer, photocatalysis has established itself as an invaluable tool for green chemistry [71]. Its reliance on cost-effective, mild reaction conditions offers a sustainable alternative to conventional synthesis [72], which explains why its use is increasing in recent years [73].

In the 2000s, there was a steady increase in the number of articles about combinatorial chemistry, as well as the emergence of biosynthesis in the 2010s and click chemistry in the following years. Combinatorial chemistry is a method for the production of chemical libraries with one common structural template [38, 74]. While its origins date back to the solid-phase peptide synthesis of the 1980s, the field has been transformed by the introduction of automated robotics for the parallel synthesis of small molecules [75]. Today, the physical combinatorial libraries are increasingly complemented by virtual libraries generated through computational chemistry, allowing for massive-scale in silico screening before physical validation [76]. Enamine REAL is one of the most remarkable examples of this methodology, with more than 13 billion compounds available for synthesis, as mentioned in Sect. “Evolution of the definition of chemical space” [50, 59].

The transition from storing full molecular structures to lightweight, machine-readable SMILES strings was the critical prerequisite for this expansion. These representations bypassed the storage constraints of earlier formats, allowing millions of molecular representations to be held in memory simultaneously. This technical advancement enabled the creation of expansive molecular repositories and the implementation of high-throughput computational workflows that were previously impossible.

Consequently, the computational power required to navigate these ultra-large libraries has necessitated a shift toward more resource-efficient screening paradigms. Several methods, such as Synthetically Accessible Virtual Inventory (SAVI), focus on optimizing compound libraries by using fragments or synthons to reduce storage space and computational load [77]. Synthon-based virtual screening has emerged as a robust alternative, applying combinatorial logic to simplify target identification [78]. By focusing on the shape and volumetric complementarity of the synthetic building blocks (or synthons) rather than the final enumerated products, researchers can identify potential bioactive hits with a fraction of the traditional computational cost. This approach effectively collapses the search space, allowing for the rapid prioritization of molecules that are likely to show biological activity upon synthesis [79–82].

Moreover, the field has taken a turn toward the use of automated laboratories for compound synthesis in an effort to reduce human error and time invested in compound synthesis [83]. The development of programming languages, such as the Chemputer platform, is designed to standardize and communicate a synthesis protocol to a computer and automate the process [84]. This convergence of resource-efficient computational mapping and programmable laboratory automation marks the beginning of an era where the exploration of the chemical universe is limited no longer by human labor or storage capacity, but by the creativity of the underlying algorithmic frameworks.

Besides combinatorial chemistry, biosynthesis is one of the most used methods to augment the size of chemical space. Natural products are the result of metabolic pathways; thus, chemists can engineer the synthesis of new molecules through the hijacking of enzymes [85]. This biocatalytic approach is particularly effective for generating structurally complex and high-molecular-weight molecules. Such compounds, which often feature multiple chiral centers and complex ring systems, represent a “natural product-like” space that is both biologically relevant and synthetically challenging to replicate through standard protocols. Asymmetric synthesis has emerged as a complementary approach, accessing similar chiral regions of chemical space through purely synthetic routes by using chiral catalysts or auxiliaries to control stereochemical outcomes [86, 87]. Together, these strategies reflect a broader recognition that stereochemical diversity is an underexplored dimension of chemical space [88, 89].

Click chemistry, introduced by Sharpless and colleagues in 2001, has seen steady growth in the literature as a strategy for expanding chemical space [90]. Its reliance on simple, commercially available building blocks allows chemists to rapidly assemble large compound collections through modular fragment coupling [91]. This compatibility with combinatorial workflows and virtual library enumeration has made click chemistry particularly useful for drug discovery applications, where the efficient synthesis of structurally diverse libraries is a central goal.

## Change in the number and rate of chemicals

To analyze the historical expansion of small and large molecules, we surveyed two major public databases. Although these repositories do not capture the totality of known chemicals given that a number of proprietary libraries remain confidential within the pharmaceutical and chemical industries, they serve as a robust proxy. Consequently, they provide a reliable reflection of the relative growth and evolutionary trends of chemical space as documented in the public domain.

### Small molecules

**Fig. 3:**
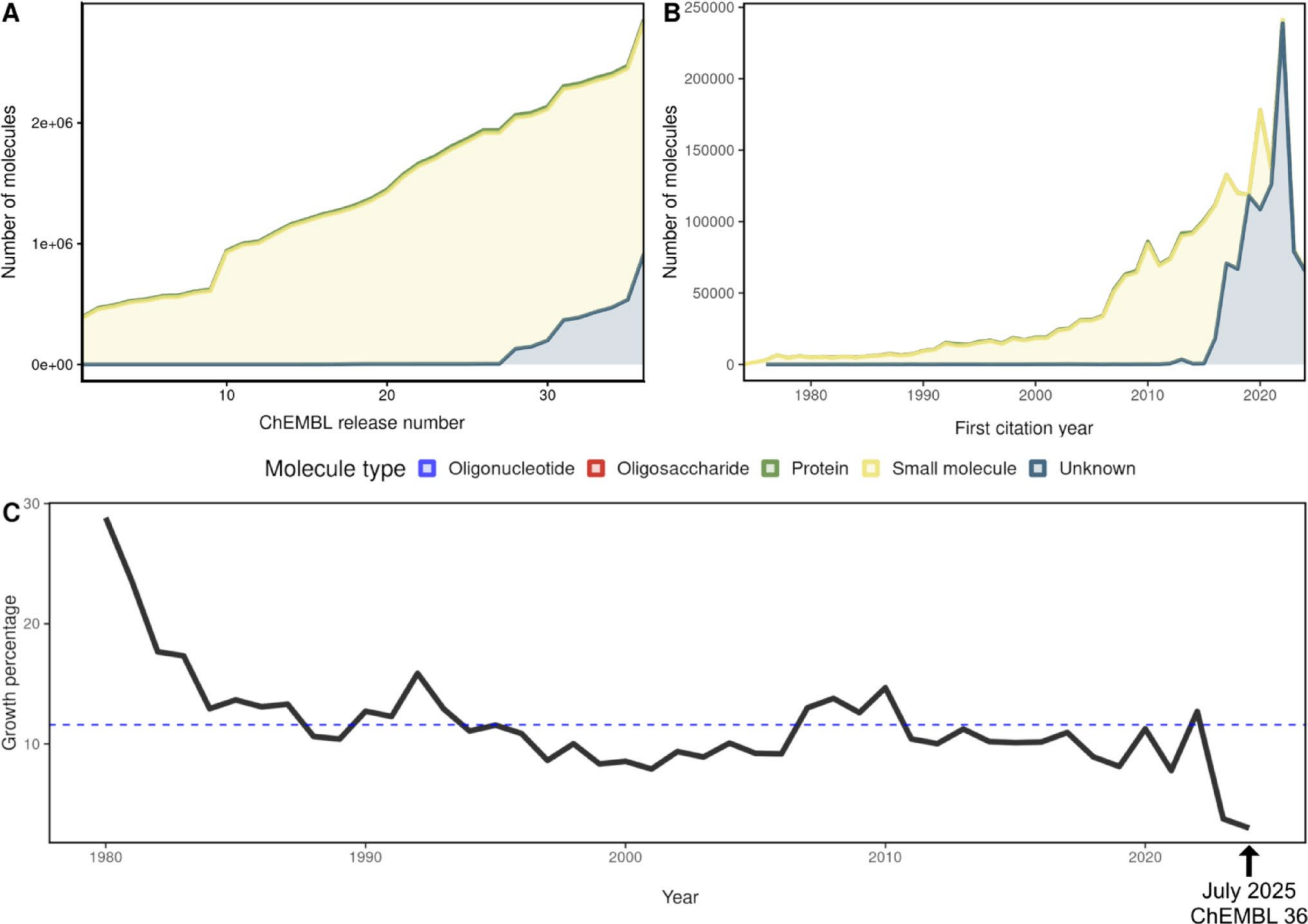
Historical expansion of ChEMBL. **A** Cumulative growth of chemical entities deposited in the ChEMBL database from its first release (October 2009) to the number 36 (July 2025). **B** Annual frequency of unique ChEMBL molecules appearing in scientific literature for the first time, reflecting the expansion of the chemical space explored by the research community. **C** Growth percentage of new molecular citations from 1980 to 2024. The solid line depicts the year-over-year percentage increase, while the dashed line indicates the mean growth rate (11.6) across the evaluated period

ChEMBL was selected as a proxy to survey the known chemical space annotated with biological activity and available in the public domain due to the comprehensive availability of its historical record, encompassing 36 sequential releases from its introduction in October 2009 to the most recent update in July 2025. As illustrated in Fig. 3A, the database shows an average compound deposition growth rate of 70,368 molecules per release. While the repository is predominantly composed of small molecules, it also includes discrete populations of oligonucleotides, oligosaccharides, and proteins (see Supplementary Figure S4 for a summary of selected drug-like continuous properties). It is worth mentioning that in recent versions of ChEMBL (28 through 36), there is a marked increase in molecules labeled as “Unknown.” However, based on their structural properties and historical composition trends, these entries are likely small molecules and were treated as such for the purposes of this analysis.

To visualize the chronological expansion of this chemical space, the “origin” of each unique chemical entity was defined as its earliest recorded year of appearance in the scientific literature, as indexed within ChEMBL. Figure 3B tracks the introduction of these molecules from 1976 to 2024. The data reveals three distinct inflection points in the volume of cited molecules, occurring in 2007, 2020, and 2022. The first citation wave is likely attributable to the maturation of high-throughput screening (HTS) technologies during the early 2000s, which enabled the simultaneous testing of thousands of compounds [92]. The subsequent waves in 2020 and 2022 may be driven by a confluence of factors, such as the global COVID-19 pandemic, the integration of AI in drug discovery efforts, and the widespread adoption of automated bioassay workflows [93–95]. Specifically, the pandemic appears to have catalyzed the adoption of HTS methodologies and accelerated data collection as the scientific community pursued effective antivirals, thereby increasing the public availability of bioactivity data on small compounds [44, 92, 95].

Despite these episodic surges, the annual growth rate has remained remarkably consistent, with an average yearly increase of 11.6% as shown in Fig. 3C. This represents a significant acceleration compared to broader historical trends; for instance, a prior study evaluating compounds reported between 1800 and 2015 identified a stable, albeit lower, exponential growth rate of 4.4% [6]. Nonetheless, the observed growth rate is close to the ideal 12.69% proposed by Restrepo in 2022 to achieve the comprehensive synthesis of the estimated possible molecules by 2050. This figure is often cited as a conceptual upper bound of 10^200^ possible molecules for entities composed of C, N, O, P, S, and halogens with a molecular weight below 1000 Da [8]. While such high-order extrapolations represent a theoretical frontier rather than a precise enumeration of synthesizable compounds, they underscore the staggering disparity between the potential chemical universe and the 2.8 million bioactive compounds currently cataloged in the public domain. This remains significant even when compared to more conservative estimates, such as those by Bohacek et al., who proposed a space of 10^60^ molecules containing up to 30 atoms. Consequently, our data suggests that the sector of the chemical universe dedicated to biological activity is currently expanding at nearly triple the rate of general chemical synthesis reported over the last two centuries [60].

#### Scaffold evolution in small molecules

While the previous section demonstrates that chemical space is expanding at an accelerated pace, prior studies suggest this growth does not necessarily translate to increased structural diversity. In 1996, Murcko and Bemis proposed the use of a shape description method to analyze common molecular shapes, defined as two-dimensional topological graphs of the molecules. After evaluating the 5120 compounds available at the Comprehensive Medicinal Chemistry (CMC) database, they found that half of the database could be described by 32 of the most frequent shapes, suggesting a remarkably low diversity among bioactive compounds [41]. A most recent example is the work by López Pérez et al., where they analyzed the molecular diversity of a set of chemical libraries (ChEMBL, DrugBank, and PubChem) and concluded that the current trend in medicinal chemistry favors the numerical expansion of chemical entities over the exploration of novel structural diversity [3].

To evaluate the structural complexity of this space, molecular diversity trends were assessed by tracking Bemis-Murcko scaffold counts across the ChEMBL version 36 historical record. The dataset was standardized by desalting and neutralizing canonical (SMILES) strings [30] prior to scaffold extraction via RDKit. Using the earliest literature citation for each compound as reference, we calculated the annual growth of the scaffold library. The Shannon Entropy (SE), a metric traditionally used to quantify species diversity [96], provides a robust framework for assessing chemical libraries. By treating unique molecular scaffolds as individual “species” within a defined chemical space, SE quantifies the structural variety and complexity of the dataset [97]. The Shannon Entropy is defined as:

$$ SE = - \mathop \sum \limits_{i = 1}^{n} p_{i} log2\left( {p_{i} } \right) $$

where* i* represents a unique Bemis-Murcko scaffold, *n* is the total number of scaffolds in the sample, and *p* is the proportion of molecules containing a specific scaffold. Higher SE values indicate a more diverse molecular “ecosystem,” while lower values suggest a restricted structural distribution.

**Fig. 4:**
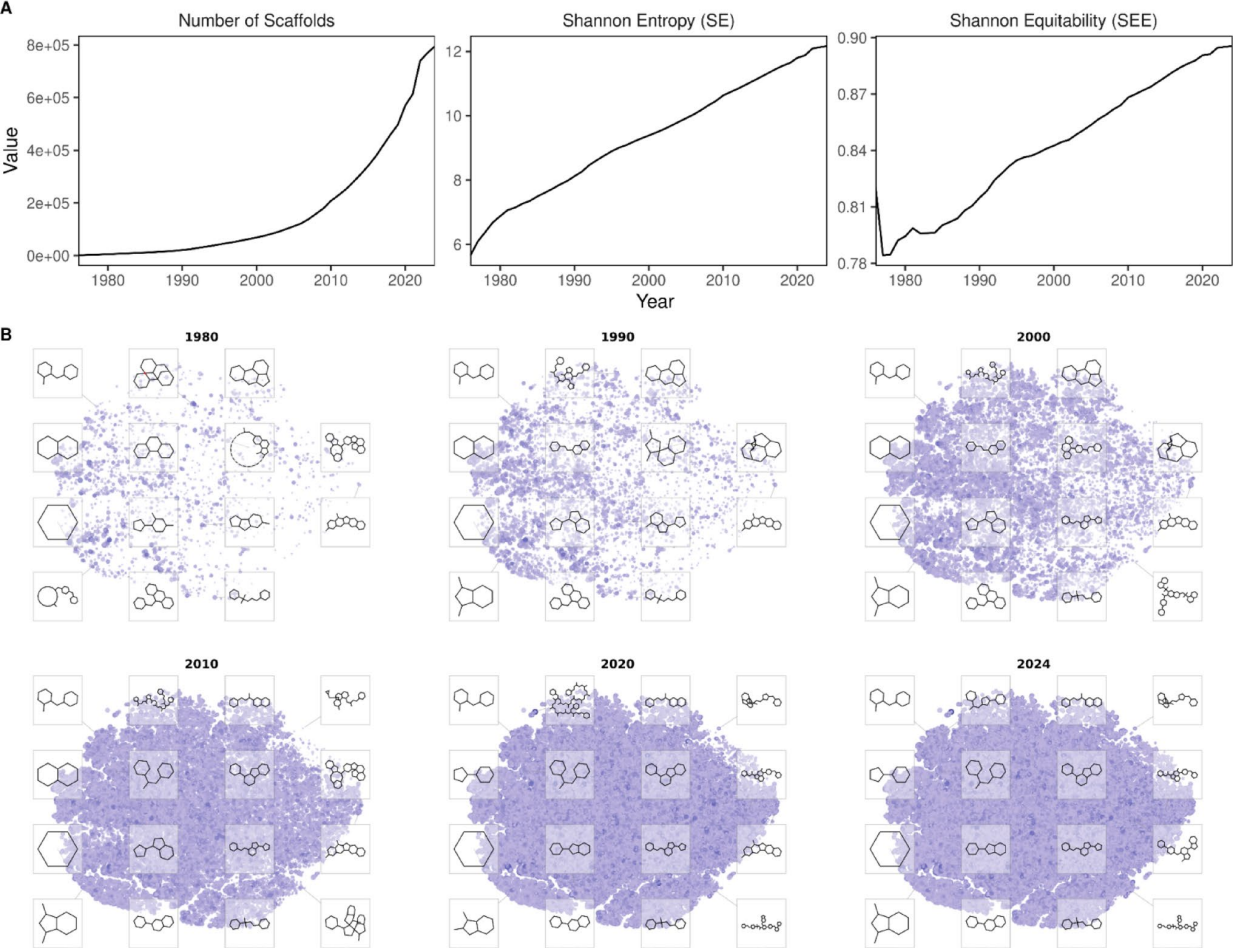
Scaffold diversity from 1976 to 2024. **A** Cumulative growth of unique Bemis-Murcko scaffolds derived from chemical entities deposited in the ChEMBL database. Longitudinal analysis of database diversity and complexity, quantified using Shannon Entropy (SE) and Shannon’s Equitability (SEE) to assess the distribution and richness of the chemical space over time. **B** Representative chemical space snapshots. High-dimensional ECFP4 fingerprints were processed via Principal Component Analysis (PCA) followed by t-distributed Stochastic Neighbor Embedding (t-SNE) to project structural similarities into two-dimensional coordinates. Node size and color intensity are proportional to the frequency of each framework within the database. Representative structural examples of selected scaffolds in diverse areas of the chemical space are shown (see also Supplementary Video 1)

Our analysis of bioactive compounds in ChEMBL reveals an increase in both the total number of scaffolds and the annual Shannon Entropy (Fig. 4A). However, a known challenge in drug discovery is the high degree of structural redundancy, where a vast majority of molecules are derived from a limited set of scaffolds. To account for this, we calculated the Shannon Equitability (SEE), also known as the “Evenness” index, which normalizes the entropy against the total number of scaffolds:

$$ SEE = SE / log\left( n \right) $$

The SEE value ranges from 0 to 1. A value approaching 0 indicates that a few highly frequent scaffolds dominate the chemical space, whereas a value of 1 represents a perfectly equal distribution of molecules across all available scaffold types. Our results demonstrate that while the SEE was initially low, it has exhibited a steady increase, approaching 0.9 in recent years. This trend indicates a significant expansion in both the absolute number of unique scaffolds and the evenness of their distribution across the chemical library. However, despite this diversification, certain molecular frameworks remain disproportionately represented. Table 2 summarizes the 10 most frequent scaffolds; benzene occupies the top rank (2%), followed by other common medicinal chemistry frameworks, including biphenyl, pyridine, indole, benzanilide, pyrrolidine, quinoline, chalcone, isoflavone, and naphthalene derivatives. This is not surprising; indeed, these groups have been previously described as “privileged structures” for drug discovery design due to their abundance in bioactive molecules [98–100].

**Table 2:** Ten most common scaffolds in ChEMBL version 36

Scaffold (Structure)	Scaffold (SMILES)	Name	Percentages^a^ (%)
	c1ccccc1	Benzene	2.08
	c1ccc(-c2ccccc2)cc1	Biphenyl	0.19
	c1ccncc1	Pyridine	0.19
	c1ccc2[nH]ccc2c1	Indole	0.17
	O=C(Nc1ccccc1)c1ccccc1	Benzanilide	0.16
	C1CCNC1	Pyrrolidine	0.13
	c1ccc2ncccc2c1	Quinoline	0.13
	O=C(/C=C/c1ccccc1)c1ccccc1	Chalcone	0.13
	O=c1cc(–c2ccccc2)oc2ccccc12	Isoflavone	0.12
	c1ccc2ccccc2c1	Naphthalene	0.12

^a^Percentage relative to the total number of molecules deposited in ChEMBL version 36

To visualize the structural evolution of the bioactive universe, we generated a series of chemical space snapshots (Fig. 4B and Supplementary Video 1). This analysis focuses on the distribution of generic frameworks (Bemis-Murcko scaffolds, where all atoms are converted to carbons and all bonds to single bonds) to emphasize the underlying molecular shapes. The mapping process began with the extraction of generic frameworks for all entities in ChEMBL release 36; these were encoded into Extended Connectivity Fingerprints diameter 4 (ECFP4) [101] to capture circular structural motifs, then a Principal Component Analysis (PCA) was performed. The resulting principal components were used as input for t-distributed Stochastic Neighbor Embedding (t-SNE) [102]. The output was used to map the frameworks into a two-dimensional coordinate system, ensuring that structurally similar scaffolds are clustered together in the visual space.

A longitudinal comparison of these snapshots reveals a marked expansion of the chemical landscape. While early maps (1980–1990) show a high concentration of molecules around a few regions, the more recent projections (2020–2024) display a significantly more dispersed distribution (Fig. 4B). This progression corroborates our earlier findings regarding the sustained growth rate and suggests that, while privileged frameworks such as benzene remain central, there has also been an expansion towards unexplored regions of the space.

### Macromolecules

So far, we have discussed the role of small molecules and a few oligomers; however, there is also a whole other universe comprising the biological polymers such as proteins, nucleic acids, and oligosaccharides. While small-molecule space is largely explored via chemical combinatorics, the biomacromolecular space follows the rules of biological evolution. We include this domain because the current evolution of chemical space is increasingly defined by the blurring of these boundaries. Modern paradigms no longer treat these as separate universes; rather, breakthroughs in AI, such as AlphaFold 3, now model the interaction of small molecules, proteins, and nucleic acids within a single unified computational framework. The integration of these transformer-based architectures has revolutionized both chemistry and structural biology [103, 104]. The disparity in data density—where only 0.1% of known proteins have solved structures compared to the nearly 3 million bioactive small molecules indexed in ChEMBL—provides critical context for the current expansion of the field.

However, there has been some research on the exploration and expansion of the peptide chemical space [105–107], nucleic acid-like molecules [108, 109], and oligosaccharides [110–112]. These efforts are frequently directed toward the “de novo” discovery of unexplored macromolecules with therapeutic potential but lacking a direct biological origin. Despite these advancements, a primary limitation is evident: the scarcity of high-resolution structural data.

**Fig. 5:**
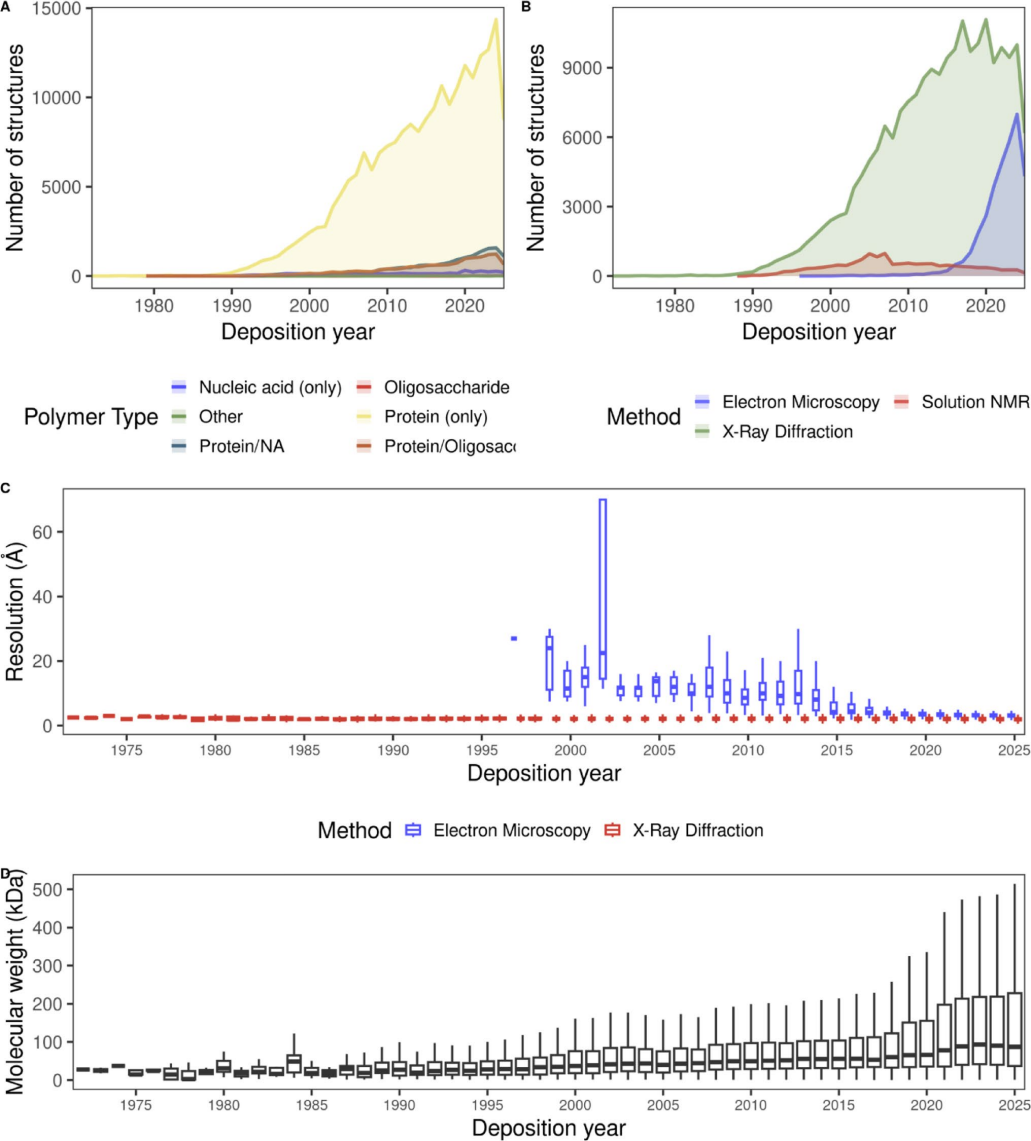
Historical records of structures deposited in the Protein Data Bank (PDB). **A** Cumulative growth of macromolecules deposited in the PDB, categorized by the three primary experimental methods: X-ray crystallography, Nuclear Magnetic Resonance (NMR), and Electron Microscopy. **B** Annual frequency of new structure depositions from 1976 to 2025. Bars are colored by polymer type. **C** Resolution distribution (in Å) for entries solved via X-ray diffraction and electron microscopy. **D** Molecular weight (kDa) distribution for the assemblies deposited in PDB through the years

The primary repository for macromolecular structures is the Protein Data Bank (PDB) [113], which, as of March 2026, holds 251,806 different experimentally solved models. Figure 5A shows the number of models deposited in PDB each year since its creation in 1971. Most of the structures are for proteins alone or in complex with nucleic acids or oligosaccharides. This data stands in stark contrast to the nearly 3 million bioactive small molecules indexed in ChEMBL, highlighting a significant disparity between chemical and structural data density in the public domain. Even more pronounced is the divergence between structural data and genomic information; the UniProtKB database [114], one of the largest databases for protein sequences, contains 203,130,941 distinct proteins in the 2026_01 release. Thus, we can roughly estimate that only 0.1% of known proteins have a solved structure. This gap could be largely attributed to the inherent difficulties of experimental characterization.

The landscape of 3D structural determination is defined by three core technologies, whose historical trajectories are summarized in Fig. 5B. X-ray diffraction has long served as the gold standard for high-resolution structures, despite the technical bottleneck of crystallization [115]. Nuclear Magnetic Resonance (NMR) serves a niche yet critical role in solving the structures of flexible molecules that elude static imaging [116]. However, the most significant recent trend is the rapid ascent of Electron Microscopy, starting in the latter half of the 2010s. This was achieved due to the widespread adoption of cryogenic Electron Microscopy (cryo-EM) and the advancements in direct electron detectors and image processing software [117]. Cryo-EM does not need macromolecule crystallization, which is an advantage over X-ray crystallography; however, one of its main limitations is the resolution. Figure 5C shows that, although at the beginning the models are of low resolution (between 10 and 60 Å), from 2018, the technique has reached almost the same resolution power as X-ray crystallography, with current models having 3 Å or even less in a few cases [118]. This has represented a huge advantage for structural biology; however, it is not enough to close the gap between small and large molecules.

Furthermore, an analysis of molecular weight distributions within the Protein Data Bank (PDB) reveals a clear trend toward the deposition of increasingly larger macromolecules. This growth is particularly pronounced over the last five years (Fig. 5C), suggesting a contemporary shift in research focus toward higher-order assemblies and complex architectures. This trend could be driven by recent breakthroughs in cryo-electron microscopy (cryo-EM), specifically in image processing and data collection, which have enabled high-resolution characterization of large complexes that were previously inaccessible to traditional structural methods [119].

Any discussion of modern structural biology must acknowledge its most significant advancement: the integration of AI through AlphaFold [104]. As previously noted, the implementation of transformer-based architectures has revolutionized both chemistry and structural biology [56]. AlphaFold, a deep-learning algorithm designed for 3D protein structure prediction from primary amino acid sequences, produces models that frequently (but not always) equal the precision of experimentally determined structures [120]. While initial iterations were limited to single polypeptide chains, the latest release, AlphaFold 3, has expanded to include multi-chain complexes, nucleic acid (RNA and DNA) chains, and attachment of oligosaccharides to peptide chains [103]. This shift has led to an unprecedented expansion of available structural data; as of this publication, the PDB includes 1,068,577 computational models, while the AlphaFold Protein Structure Database hosts over 200 million predictions, covering a significant part of proteins in UniProt [121]. Furthermore, the field is now pivoting toward de novo protein design, where generative models are used to engineer entirely novel proteins with no known biological origin [122, 123]. These advancements are, without question, the primary drivers of the current expansion within the macromolecular structural space.

## Journals chronicling the development of chemical space and molecular modeling

**Fig. 6:**
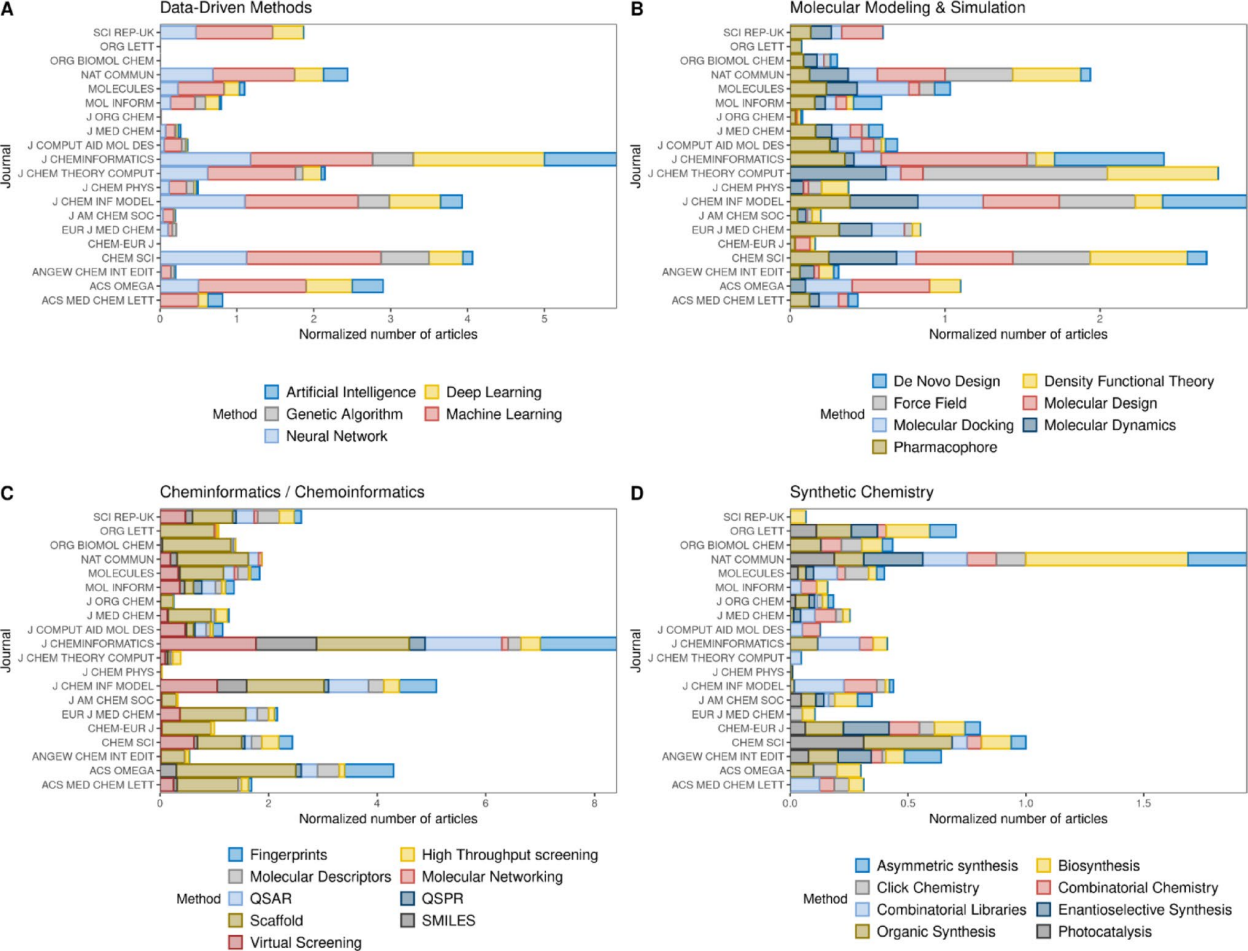
Publication landscape of the top 20 journals in Chemical Space research. The histograms illustrate the volume of articles published per journal, categorized by four primary themes: **A** Data-Driven Methods, **B** Molecular Modeling and Simulations, **C** Cheminformatics/Chemoinformatics, and **D** Synthetic Chemistry. Article number was normalized by dividing the number of articles containing each keyword between the number of years since the journal’s first issue

In this section we would like to highlight some journals that have contributed to the definition and study of chemical space. This bibliometric analysis serves as a quantitative proxy for the field’s evolution, as the growth in specialized literature directly mirrors the maturation of the underlying algorithms and the expansion of the chemical datasets they describe. Figure 6 presents the top 20 journals in the field, defined as the titles with the highest number of articles containing the “chemical space” keyword, according to Web of Science records normalized by the number of years since each journal’s first issue.

The most conspicuous pattern across Fig. 6A and C is the prominence of the *Journal of Cheminformatics*, which records among the highest annualized publication rates in both panels. Launched in 2009, this outcome confirms that its contribution to chemical space research reflects genuine thematic productivity rather than accumulated volume [124].

The *Journal of Chemical Information and Modeling* maintains a consistent and broadly distributed presence across Fig. 6A–C, reflecting its role as the field’s primary and longest-standing publication outlet for cheminformatics, molecular modeling, and data-driven approaches. Originally established in 1960 as the *Journal of Chemical Documentation*, the journal underwent two subsequent name changes mirroring the evolution of its scientific scope: in 1975 it became the *Journal of Chemical Information and Computer Sciences*, and in 2005 it was renamed *Journal of Chemical Information and Modeling* [125]. Its representation across all three computational panels is consistent with its enduring centrality to the field: a journal that has defined and accompanied the discipline since well before “machine learning” or “deep learning” entered the computational chemistry lexicon.

The case of *Molecular Informatics* warrants a methodological caveat. The journal has operated under its current name only since 2010, having previously been published as *QSAR and Combinatorial Chemistry* since 1982 [126], meaning that a substantial portion of its historical contribution to the field remains uncaptured in the bibliometric query, likely underestimating its true weight particularly in Fig. 6C.

The presence of broad-scope venues such as *Journal of Medicinal Chemistry*, *Journal of the American Chemical Society*, and *Angewandte Chemie International Edition* in Fig. 6A and B reflects the progressive integration of computational methods into experimental drug discovery pipelines. These journals do not publish cheminformatics as a primary focus but increasingly incorporate it as a methodological component of experimentally driven work, which accounts for their modest yet consistent representation. Journals such as *Nature Communications* and *ACS Omega*, both characterized by high publication volume and relatively recent launches, emerge with competitive annualized rates particularly in Fig. 6C and D, underscoring the extent to which multidisciplinary and mega-journals have become relevant dissemination channels for chemical space research.

The comparatively modest representation of the *Journal of Computer-Aided Molecular Design* (JCAMD) in Fig. 6A reflects deliberate thematic scope rather than limited productivity. Established in 1987, JCAMD has served for nearly four decades as a dedicated venue for structure-based and simulation-driven approaches to molecular design [127], a focus more consistent with its presence in Fig. 6B than with the data-driven methods category of Fig. 6A. Under the long-standing editorial stewardship of Dr. Terry R. Stouch as Senior Editor-in-Chief, the journal cultivated a rigorous tradition in computational drug design with particular emphasis on methodological transparency and error assessment, contributions that shaped the field well beyond its bibliometric footprint. Its lower count in Fig. 6A should therefore be read alongside Fig. 6B, where JCAMD’s footprint more accurately reflects its historical and ongoing influence.

Finally, the reference to Supplementary Table S1 serves as a necessary corrective to the overall reading of Fig. 6. The journals represented here are predominantly established titles with decades of accumulated output. The growth frontier of chemical space research is progressively migrating toward newer venues, such as *Digital Discovery* (launched 2022) and other nascent journals focused on AI applications in chemistry. By virtue of their youth, these titles do not yet produce expressive bars in retrospective bibliometric analyses but are well-positioned to substantially reshape this publication landscape over the coming decade.

## Conclusions and future directions

Historically, the concept of chemical space has evolved from an abstract theoretical ensemble of all possible molecules into a pragmatic, computational framework used to navigate molecular diversity. It can be considered that the birth of chemical space began as the first chemical compounds were made although the systematic study of chemical space began with the start of the first compounds collections and chemical system [128]. From a historical point of view, a systematic literature search revealed that the term “chemical space” itself (as used to this date) was first published in the literature in 2004 although there are several previous studies dating back to the 1970s that have studied the relationships between compounds in a descriptor space.

As the chemical space expands and diversifies, a number of computational strategies have emerged and evolved in response to the increasing number of chemicals that populate the vast chemical universe. Central to this evolution is the integration of AI, which has fundamentally redefined our capacity to navigate the vast multidimensionality of chemical space. Architectures such as AlphaFold have served as primary drivers in the expansion of macromolecular structural space, providing biologically relevant models. Such tools allow us to explore the unknown chemical space where experimental methods have not reached yet.

The history and account of chemical space and molecular modeling has been documented across at least 20 well-established peer-reviewed journals. New technologies and the fast-growing number of chemical compounds and the methodologies to explore the vast chemical universe has led to the establishment of new peer-reviewed journals that incorporate AI and machine learning as key components to their scope. This reflects the field maturation, as shown by the prevalence of journals specifically dedicated to computational methods such as the *Journal of Computational Aided Molecular Design* and the *Journal of Chemical Information and Modeling.* This provides a platform for theoretical chemistry and practical pharmaceutical application, fostering a community focused on the rational design of small molecules.

Future directions will continue to be influenced by data availability and computational scale [129]. It is anticipated to see an increase in the computational methods to handle large and ultra-large chemical libraries not only for drug discovery applications but for other chemical domains; incremental use of generative models to enumerate unexplored but relevant areas of chemical space (expected to see a continued increase in the chemical/scaffold diversity of the newly generated libraries). However, it is important to keep a balance between the use of traditional or classical approaches and the newest trends or methodologies based on the goals of the projects and not “AI-hype” driven applications.

## Supplementary Information

Below is the link to the electronic supplementary material.

Supplementary Material 1

Supplementary Material 2

## Data Availability

All reported data is included and discussed in the manuscript. Supplementary Video 1 is available in Zenodo under the following doi: https://doi.org/10.5281/zenodo.19502840. The source code and data used in this article is deposited in https://github.com/aylindmm/The-r-evolution-of-chemical-space.
