# Supplementary material for: The (r)evolution of chemical space and molecular modeling: a time-resolved perspective
Source: J Comput Aided Mol Des. 2026 Jun 1;40(1):136. doi: 10.1007/s10822-026-00850-1 (PMC13226435; doi:10.1007/s10822-026-00850-1)
Supplement: Supplementary file 2 — Supplementary Material 2 [file 10822_2026_850_MOESM2_ESM.pdf]

SUPPORTING INFORMATION

The (r)evolution of chemical space and molecular modeling: A time-resolved perspective

Aylin Del Moral-Morales,[1,*] Francisco L. Feitosa,[2] Carolina Horta Andrade,[2] José L. Medina-Franco[1,*]

[1] DIFACQUIM Research Group, Department of Pharmacy, School of Chemistry, Universidad Nacional Autónoma de México, Avenida Universidad 3000, Mexico City 04510, Mexico

[2] Laboratory for Molecular Modeling and Drug Design (LabMol), Faculty of Pharmacy, Universidade Federal de Goiás, Rua 240, 406, Goiânia, Goiás, 74605-220, Brazil

Contents

		Page
**Figure S1**	Bibliometric landscape of "Chemical Space" literature.	S2
**Figure S2**	Annual publication trends from 1995 to 2025 for articles related to Data-Driven Methods, Molecular Modeling and Simulations, and Cheminformatics / Chemoinformatics.	S3
**Figure S3**	Annual publication trends from 1995 to 2025 for peer-reviewed articles related to chemical space and indexed with keywords related to Synthetic Chemistry.	S4
**Figure S4**	Distribution of selected drug-like continuous properties of molecules deposited in CHEMBL, release 36.	S5
**Table S1**	Representative new journals publishing papers covering advances in molecular modeling, chemical space, and related subjects.	S6

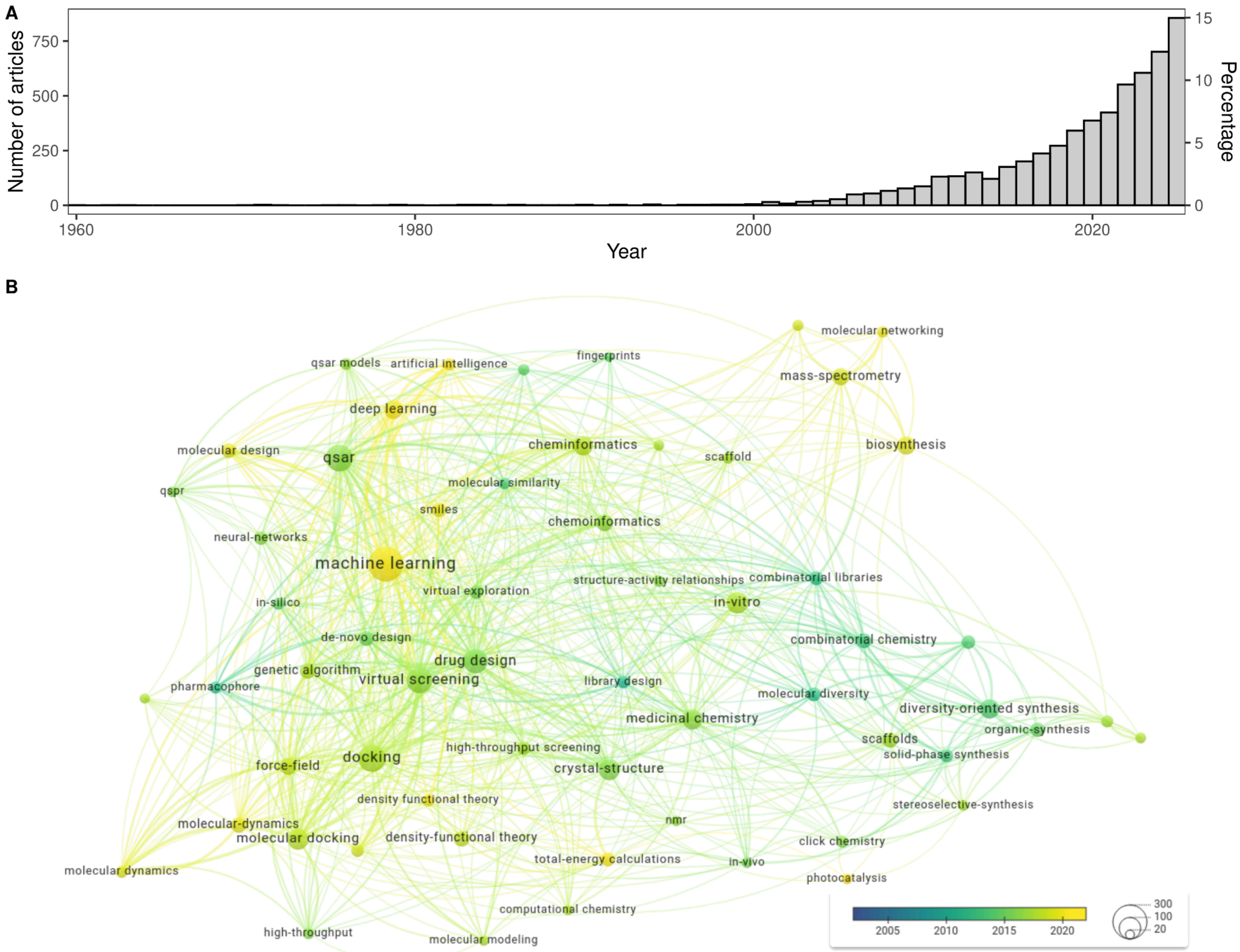

Figure S1. Bibliometric landscape of "Chemical Space" literature. A) The histogram shows the number of articles published containing the keyword “Chemical space” from 1960 to 2025, retrieved from Web of Science. B) Co-occurrence network of keywords generated via VOSviewer. Node diameter is proportional to citation frequency; edges indicate the strength of association between terms found within the same publication records.

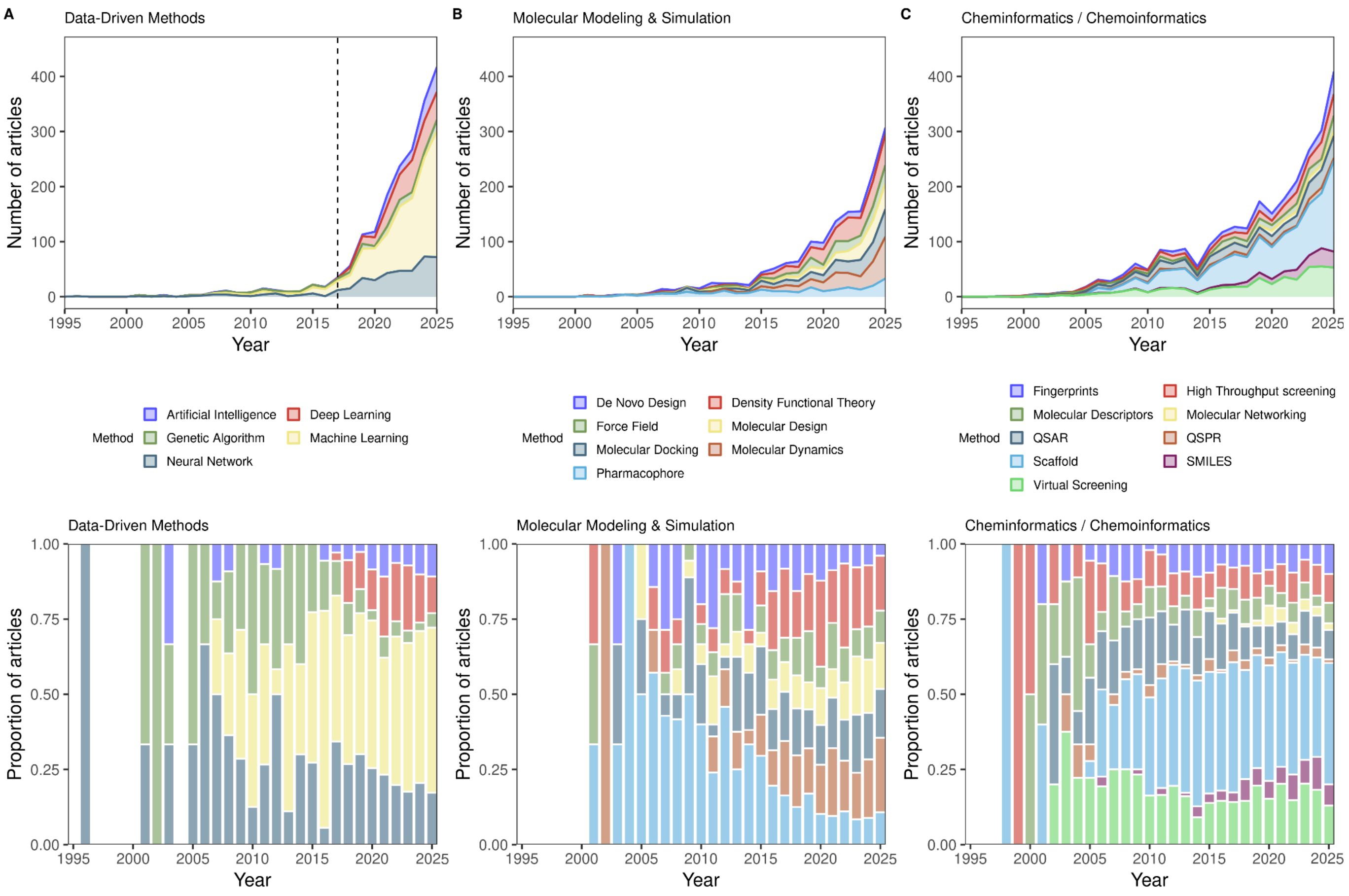

Figure S2. Annual publication trends from 1995 to 2025 for articles indexed with keywords related to C) Data-Driven Methods (the dashed line indicates the publication of "Attention Is All You Need" in 2017), D) Molecular Modeling and Simulations, and E) Cheminformatics / Chemoinformatics. The upper panel shows the number of articles published with each keyword, and the lower panel shows the proportion of articles published each year.

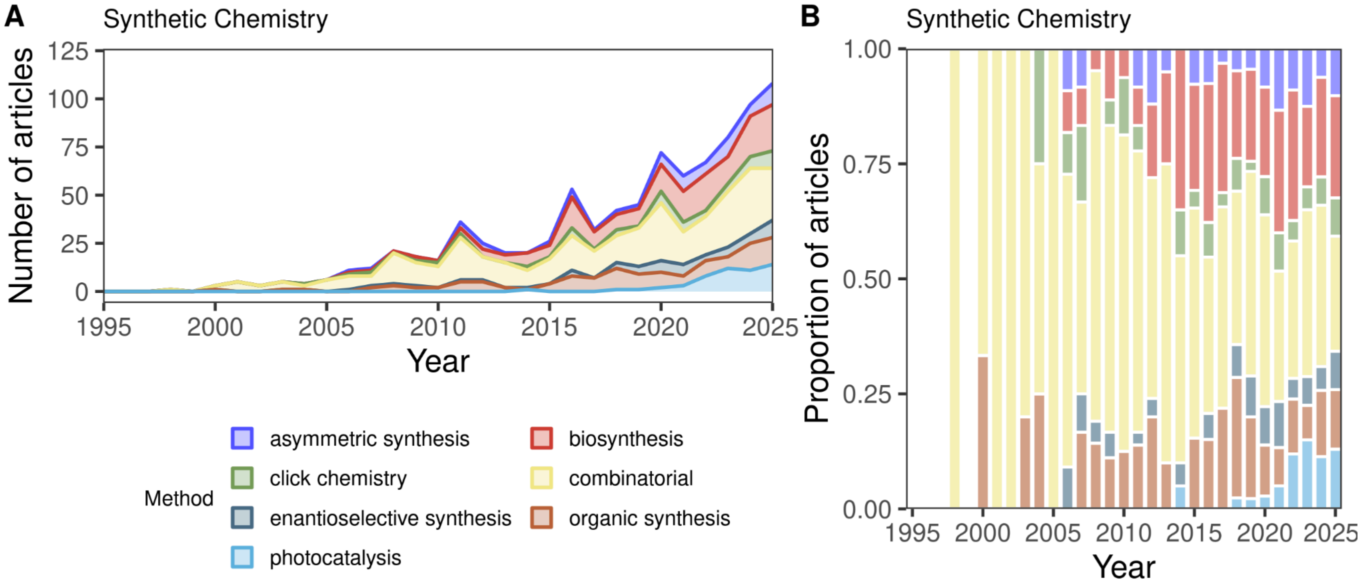

Figure S3. Annual publication trends from 1995 to 2025 for peer-reviewed articles related to chemical space and indexed with keywords related to Synthetic Chemistry. A) The number of articles published with each keyword. B) The proportion of articles published each year.

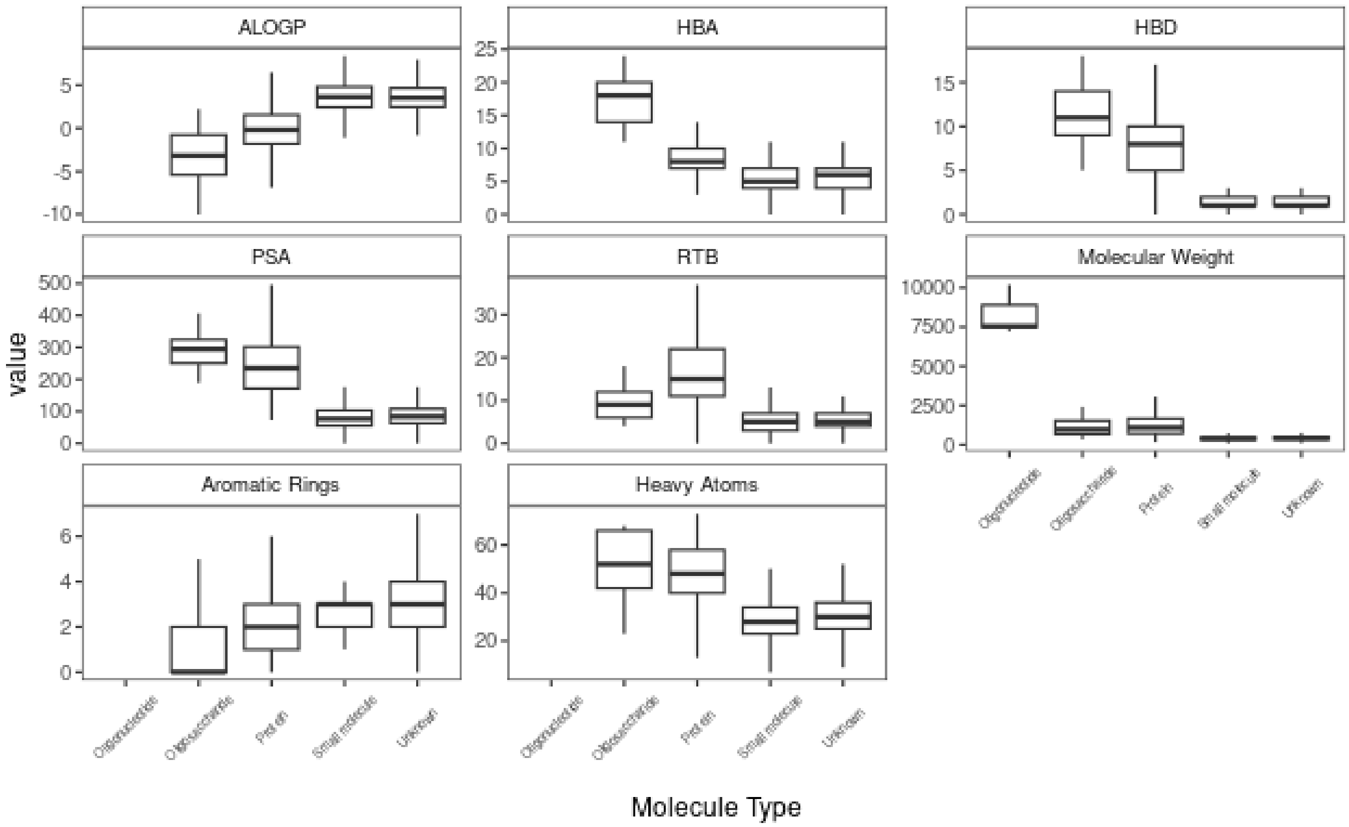

Figure S4. Distribution of selected drug-like continuous properties of the 2,878,135 molecules deposited in CHEMBL, release 36.

Table S1. Representative new journals publishing papers covering advances in molecular modeling, chemical space, and related subjects.

**Journal** [a]	**Established** [b] (year) Published its first content	**Scope**[c]
AI Chemistry	2026	"... development and application of artificial intelligence in all areas of fundamental and applied chemical sciences…"
Artificial Intelligence Chemistry	2023	"The journal will broadly cover all areas of chemistry where artificial intelligence (AI) and machine learning approaches are used."
Artificial Intelligence in the Life Sciences	2021	"A forum for theoretical and methodological advances and practical applications of Artificial Intelligence (AI) in the broader life science area"
Digital Discovery	2022	"Welcomes both experimental and computational work on all topics related to the acceleration of discovery such as screening, robotics, databases and advanced data analytics, broadly defined, but anchored in chemistry."
Frontiers in Drug Discovery (section: In silico Methods and Artificial Intelligence for Drug Discovery)	2021	"Multidisciplinary journal focused on the discovery and development of new drugs and therapies." "The In silico Methods and Artificial Intelligence for Drug Discovery section is dedicated to publishing research focused on advancing computer-aided drug design and discovery through theoretical and experimental studies."
Nature Machine Intelligence	2019	"... a wide range of topics in machine learning, robotics and AI…."

[a]In alphabetical order. [b]Year of establishment/launched. In this manuscript a journal is considered young/new if it was launched in 2019 to date. [b]Part of the scope as stated in the published web-site.
